# Comparative Study of Bioactivity and Safety Evaluation of Ethanolic Extracts of *Zanthoxylum schinifolium* Fruit and Pericarp

**DOI:** 10.3390/molecules26195919

**Published:** 2021-09-29

**Authors:** Jun Gu Kim, Jae Jung Lim, Ji Sang You, Hyeok Jun Kwon, Heung Bin Lim

**Affiliations:** Department of Industrial Plant Science and Technology, Chungbuk National University, Cheongju 28644, Korea; wnsrndlsn@chungbuk.ac.kr (J.G.K.); ijj0201@naver.com (J.J.L.); r9510@naver.com (J.S.Y.); gurwns304@naver.com (H.J.K.)

**Keywords:** *Zanthoxylum schinifolium*, fruit, pericarp, safety evaluation, antimutagenic activity, antioxidant activity, anti-inflammatory activity

## Abstract

The fruit and pericarp of *Zanthoxylum schinifolium* (ZS) have been used in traditional medicine; however, few studies have characterized ZS fruit and pericarp. Therefore, in the present study, we evaluated the safety of ZS fruit (ZSF) and pericarp (ZSP) extracts and compared their bioactivity. To evaluate the safety of ZSF and ZSP, mutagenicity, cytotoxicity, and oxidative stress assays were performed and nontoxic concentration ranges were obtained. ZSP was found to be superior to ZSF in terms of its antimutagenic, antioxidant, and anti-inflammatory activities. In the S9 mix, the mutation inhibition rate of ZSP was close to 100% at concentrations exceeding 625 µg·plate^−1^ for both the TA98 and TA100 strains. ZSP exhibited efficient DPPH (IC_50_ = 75.6 ± 6.1 µg·mL^−1^) and ABTS (IC_50_ = 57.4 ± 6 µg·mL^−1^) scavenging activities. ZSP inhibited the release of cytokines, involved in IL-1β (IC_50_ = 134.4 ± 7.8), IL-6 (IC_50_ = 262.8 ± 11.2), and TNF-α (IC_50_ = 223.8 ± 5.8). These results indicate that ZSP contains a higher amount of biochemicals than ZSF, or that ZSP contains unique biochemicals. In conclusion, for certain physiological activities, the use of ZSP alone may be more beneficial than the combined use of ZSF and ZSP.

## 1. Introduction

Modern society has long been combating diverse diseases, and research on treatments for such conditions has been continuously promoted. At present, humans are readily exposed to chronic disorders, such as myocardial infarction, cancer, and diabetes, which have been proposed to be major causes of death worldwide. To eradicate these chronic diseases, the modern view of therapy is changing from treatment-centered management to preventive management [1]. In this context, diet is in the spotlight as a disease preventive approach, and functional foods, which contain bioactive ingredients that nourish and benefit the body, have attracted much attention [2]. Health functional foods are products manufactured and processed from ingredients or raw materials useful for the human body to obtain health benefits, such as nutrition or biological effects. Naturally growing plants have been widely used as raw materials to produce health functional foods because of their various bioactivities, including anti-cancer effects, which are considered the most important in modern times [3]. Traditional medicinal plants have historically been considered safer than commercial drugs; however, safety evaluations must be performed.

Mutation is a permanent change in the nucleotide sequence of living DNA, which may be passed on to the daughter cells through DNA replication and cell division [4]. In general, mutations exert detrimental effects on individuals, because each gene is typically evolved to function the best in its current state [5]. Some natural products or their overdoses may induce mutagenesis and are known to act as carcinogens. Simultaneously, however, natural products can also act as anti-mutagens. The importance of mutagenicity and anti-mutagenicity evaluations of natural products is increasing, because mutagens are involved in the initiation and progression of several human diseases, including cancer [6].

Reactive oxygen species (ROS) is a generic term for molecules containing unstable oxygen, such as peroxide, superoxide, and hydroxyl ions, which are free radicals with high reactivity toward other organic substances [7]. ROS are generated by different endogenous and exogenous factors. Under ROS deficiency, cell function may be dysregulated, whereas under excess ROS accumulation, mutations may be induced following their reaction with a gene or protein in the cell, leading to the expression of cancer-related traits. Therefore, the homeostasis of free radicals must be ensured in the body [8]. ROS levels within cancer cells are higher than those in other normal cells [9]. However, cancer cells also produce antioxidant enzymes to prevent excess ROS accumulation because these radicals can lead to cancer cells’ death. Therefore, it is important to search for substances that block ROS production in normal cells as well as for natural substances that specifically generate ROS in cancer cells [9,10,11].

Inflammation is an in vivo response occurring in specific tissues that are damaged or infected [12]. Although immune activation is vital for protection against infection or disease, prolonged hyperactivation can damage healthy tissues. Therefore, the immune system activated by foreign elements must return to its prior state when its role is finished [13]. This immune regulation is mediated by signaling molecules known as cytokines, and the regulation of their expression is crucial. Cytokine overproduction can lead to cytokine storms, which are the major cause of severe infections, such as Spanish flu, avian flu, and acquired immunodeficiency syndrome [14,15,16,17]. Therefore, research on natural products that can treat cytokine storms is underway, and natural products without side effects are being actively searched for [18]. Typically, plants are considered the primary source of natural substances for disease treatment because they produce secondary metabolites, such as alkaloids, terpenoids, phenolics, and flavonoids [19]. There are various techniques that have been used to extract beneficial compounds from plants. Traditional methods include solvent extraction and cold-pressing, while innovative methods utilize ultrasonic-assisted extraction, microwave-assisted extraction, pressurized liquid extraction, and supercritical fluid extraction [20]. Drying and grinding of plant material is often performed to increase the extraction yields obtained from all extraction methods [20].

*Zanthoxylum schinifolium* (ZS) of the Rutaceae family mainly grows in Korea and China. ZS fruits are primarily used as the raw material for producing oil, and ZS pericarp is used as a spice or medicinal product [21]. ZS is called Jincho in Dongui Bogam and has been recorded as producing a warm effect, having a spicy taste, and being poisonous. However, it can cure leprosy, strengthen teeth, and prevent hair fall. Moreover, it can brighten the eyes, relieve stomach ache, and prevent dysentery caused by cold [21,22,23,24]. There are several phytochemical compounds that are obtained from ZS and possess beneficial bioactivities. For example, the anti-KSHV activity of megastigmane sesquiterpenoids; NF-κB inhibitory activity of glycosides and alkaloids; radio-sensitizing effects of 4-quinolinone derivatives; as well as the anticancer activity, apoptogenic activity, monoamine oxidase inhibitory, and antiplatelet aggregation activities of coumarins derived from ZS have been investigated [21,22,23,24,25,26,27,28]. Additionally, the vascular smooth muscle proliferation inhibitory effect, antioxidant, cytotoxicity, antibacterial, anti-melanogenic, anti-inflammatory, and insecticidal activities have been reported in ZS extracts [29,30,31,32,33].

Most previous studies on ZS focused on oil extracts, which contain non-polar substances. However, few studies have assessed ZS ethanol extracts, which contain both polar and non-polar substances, and the safety of ZS remains to be evaluated. Although *Z. schinifolium* fruit (ZSF) and pericarp (ZSP) are commercially used separately, they were combined for use in experiments in previous studies. In the present study, the mutagenicity of ZS extracts was evaluated for the first time. In addition, the safety of ZSF and ZSP was measured and their bioactivities, including antimutagenic, antioxidant, and anti-inflammatory effects, were compared.

## 2. Results and Discussion

### 2.1. ZS Extract Yield

Dried ZSF and ZSP products (300 g) were extracted with 70% ethanol for 3 days to obtain yields of 32.88 g and 76.74 g, respectively (Table 1). The combined yield of ZSP and ZSF has been reported to be 34.1% using a similar method, consistent with our results [34]. The yield for ZSP was previously reported to be 8% using ultrasonic methanolic extraction [24]; while the ZS root yielded 1% with methanol and 5% with 95% ethanol [22,28]; ZS stem yielded 2.3–4.4% with methanol [21,23,27], and ZS leaves yielded 12–22.7% with 80% methanol [25,26]. Extraction is the step of recovering and separating compounds from plant material and the extraction solvent, which is composed of water and organic solvents, allows for the simultaneous extraction of compounds soluble in water and organic solvents [35]. When comparing hot water and ethanol extraction, the higher the proportion of organic solvents, such as ethanol, the higher the amount of extracted biochemical components, such as essential oil components, polyphenols, triterpenoids, and steroids [36]. In contrast to previous studies, our results suggest that 70% ethanol was the most effective extraction method for ZS compounds. Although innovative methods to extract bioactive compounds from plants are continuously being developed, further research is required, especially in the application of supercritical CO_2_ or subcritical CO_2_ extraction [20].

### 2.2. ZS Extract Safety

#### 2.2.1. Mutagenicity

**Figure 1:**
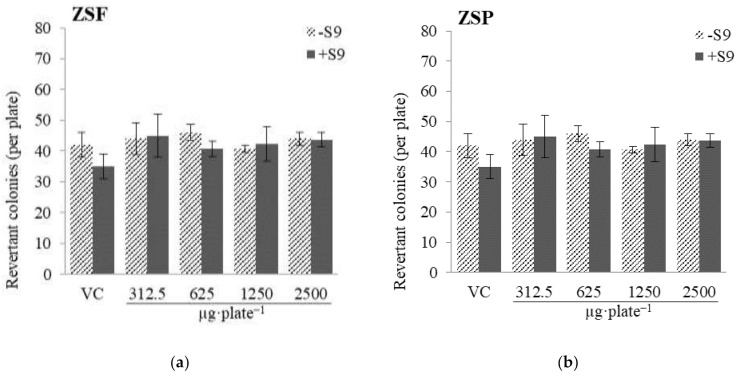
Results of mutagenicity test of *Zanthoxylum schinifolium* extracts ((**a**) ZSF: *Z. schinifolium* fruit extract, (**b**) ZSP: *Z. schinifolium* pericarp extract) in *Salmonella typhimurium* TA98, with or without the S9 mix. In the positive control, the number of revertant colonies are 267 ± 12 and 785 ± 33, with or without the S9 mix. The S9 mix consists of the rat liver S9 fraction and other co-factors. Data are expressed as the mean ± SD of triplicate experiments. VC, vehicle control (DMSO).

**Figure 2:**
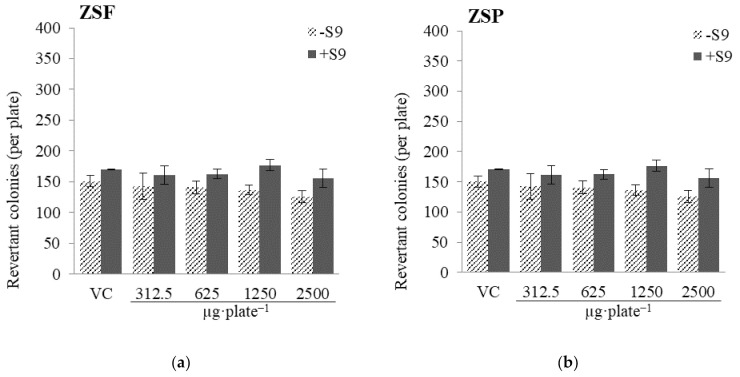
Results of mutagenicity test of *Zanthoxylum schinifolium* extracts ((**a**) ZSF: *Z. schinifolium* fruit extract, (**b**) ZSP: *Z. schinifolium* pericarp extract) in Salmonella typhimurium TA100, with or without the S9 mix. In the positive control, the number of revertant colonies are 1025 ± 37 and 753 ± 21, with or without the S9 mix. The S9 mix consists of the rat liver S9 fraction and other co-factors. Data are expressed as the mean ± SD of triplicate experiments. VC, vehicle control (DMSO).

The Ames test is an experiment to verify substances that can cause genetic modification in somatic and germ cells, and it has been widely used in initial screening during new drug development [37]. Mutagens, such as radiation, ultraviolet light, chemicals, and endogenous substances, are highly reactive to DNA and can damage it, leading to cancer progression [38]. Genotoxicity evaluations are critical, given the possible existence of mutagens among the vast resources in nature [39,40]. Here, to investigate the mutagenicity of ZS extracts, a mutagenicity assay was performed using *Salmonella typhimurium* TA98 and TA100 strains. Briefly, different concentrations (312–2500 µg·plate^−1^) of ZS extracts were processed in the presence or absence of a metabolic enzyme system (S9 mix; Figure 1; Figure 2). In the case of TA98, the number of revertant colonies in the vehicle control was 42 ± 4 without the S9 mix and 35 ± 4 with the S9 mix and the number of revertant colonies in the positive control containing the mutagen was 785 ± 33 without the S9 mix and 267 ± 12 with the S9 mix. These results indicate that the mutant system functioned normally. There was no significant increase in the number of revertant colonies (with or without the S9 mix) in ZS extract samples of different concentrations compared with that in the vehicle control (*p* < 0.05). In the case of TA100, the number of revertant colonies in the vehicle control was 151 ± 9 without the S9 mix and 170 ± 1 with the S9 mix, and the number of revertant colonies in the positive control was 753 ± 21 without the S9 mix and 1025 ± 37 with the S9 mix. There was no significant increase in the number of revertant colonies (with or without the S9 mix) in ZS extract samples of different concentrations compared with that in the vehicle control (*p* < 0.05). As a safety evaluation, the in vitro mutagenicity of ZS extracts was reported for the first time in the present study. Our results suggest that ZSF and ZSP extracts or their liver metabolites do not act as mutagens at concentrations up to 2500 µg·plate^−1^.

#### 2.2.2. Cytotoxicity

**Figure 3:**
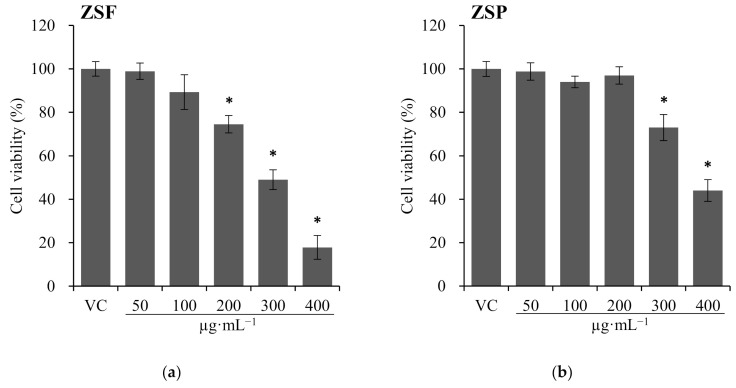
Effects of *Zanthoxylum schinifolium* extracts ((**a**) ZSF: *Z. schinifolium* fruit extract, (**b**) ZSP: *Z. schinifolium* pericarp extract) on WI-38 cells viability for 24 h. Data are expressed as the mean ± SD of triplicate experiments. * Significant difference compared with the vehicle control group (*t*-test, * *p* < 0.0201). VC, vehicle control (DMSO).

**Figure 4:**
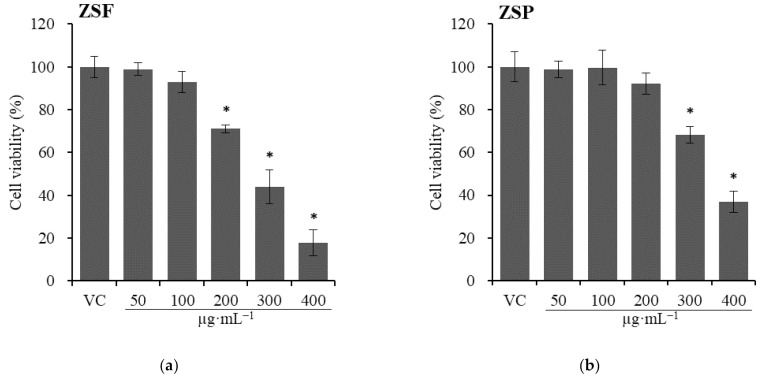
Effects of *Zanthoxylum schinifolium* extracts ((**a**) ZSF: *Z. schinifolium* fruit extract, (**b**) ZSP: *Z. schinifolium* pericarp extract) on U-937 cells viability for 24 h. Data are expressed as the mean ± SD of triplicate experiments. * Significant difference compared with the vehicle control group (*t*-test, * *p* < 0.0073). VC, vehicle control (DMSO).

Cytotoxicity assays measure the ability of materials such as chemicals, natural toxins, or immunomediators to cause cell death. Cytotoxicity assays are an important step in the evaluation of biomedical applications of plant materials and have been widely employed in basic research and drug development to screen the libraries for toxic compounds [41,42]. Here, the cytotoxicity of ZS extracts was assayed against WI-38 and U-937 cells, and the results are shown in Figure 3; Figure 4. The WST assay was performed because the neutral red uptake (NRU) assay is not suitable for U-937 cells, which are floating cells. In the case of WI-38 cells, compared with that of the vehicle control, the IC_50_ value of 200 µg·mL^−1^ ZSF (IC_50_ = 279.3 µg·mL^−1^) and 300 µg·mL^−1^ ZSP (IC_50_ = 380.5 µg·mL^−1^) differed significantly (*p* < 0.0020). In the case of U-937 cells, compared with that of the vehicle control, the IC_50_ value of 200 µg·mL^−1^ ZSF (271.6 µg·mL^−1^) and 300 µg·mL^−1^ ZSP (388.2 µg·mL^−1^) differed significantly (*p* < 0.0003). In previous study, the cytotoxicity of ZS extract was assayed but the reported profile differs from the results of cytotoxicity assays in the present study. At concentrations lower than 300 µg·mL^−1^, no toxicity is observed in the ZS methanolic extract [32]. The single use of ZSF and ZSP, as well as the use of other cell lines, may be attributed to this difference. In addition, the high reactivity of the NRU assay may be attributed to this difference, as the NR reagents are sensitive to lysosomal damage [43]. This is the first study to investigate and compare the cytotoxicity between ZSF and ZSP extracts, and the ZSP extract was found to possess a wider range of therapeutic window than ZSF (Figure 3).

#### 2.2.3. Intracellular ROS Generation

**Figure 5:**
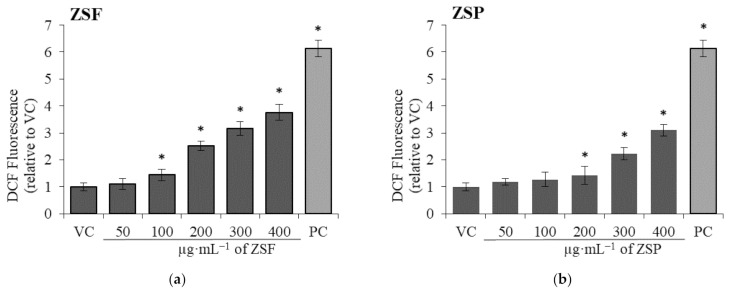
Intracellular reactive oxygen species (ROS) generation induced by *Zanthoxylum schinifolium* extracts ((**a**) ZSF: *Z. schinifolium* fruit extract, (**b**) ZSP: *Z. schinifolium* pericarp extract) in WI-38 cells. Data are expressed as the mean ± SD of triplicate experiments. * Significant difference compared with the vehicle control group (*t*-test, * *p* < 0.0486). VC, vehicle control (DMSO). PC, positive control (500 µM H_2_O_2_).

This assay was performed to determine whether the ZS extracts induced oxidative stress via ROS generation in WI-38 cells. ROS generation in normal cells following ZS extract treatment is shown in Figure 5. Dichlorofluorescein (DCF) fluorescence obtained through the oxidation of dichlorodihydrofluorescein diacetate (DCFDA) was used to quantify ROS. The cells were treated with different concentrations of ZS extracts, and the results were expressed as relative values (%) to the vehicle control; 500 µM H_2_O_2_ was used as the positive control. ROS production significantly differed between treatment with 100 µg·mL^−1^ ZSF extract and vehicle control, with ROS levels increasing in a concentration-dependent manner. Likewise, ROS production significantly differed between treatment with 200 µg·mL^−1^ ZSP extract and vehicle control, with ROS levels increasing in a concentration-dependent manner. At high concentrations, natural chemicals induce intracellular ROS generation, leading to oxidative stress in the mitochondria or endoplasmic reticulum and ultimately resulting in cell death via apoptosis and/or necrosis [44,45]. The observed cytotoxicity of the ZS extracts against WI-38 cells may be due to ROS-mediated oxidative stress or toxicity of the extract itself. Several phytochemicals, including sesquiterpenoids, glycosides, alkaloids, and coumarins, induce cell death via ROS generation [46,47,48,49,50,51,52,53,54,55]. Therefore, ZS extracts, containing schinifolenol A, zanthoxyloside A, norchelerythrine, and auraptene, have the potential to induce oxidative stress-induced cell death [22,23,26]. Dimethyl sulfoxide (DMSO), which was used as a vehicle control, is an effective antioxidant that may influence ROS generation [56,57]. However, we used diluted DMSO (<1%) in this experiment, which is considered to have little or no effect on the ROS production [57]. In a previous study, ROS production was measured following treatment with ZS oil extracts [58]. The present study is the first to measure intracellular ROS production by comparing ZSF and ZSP extracts, and our results suggest that at the same concentration, ZSP extract induces less oxidative stress than ZSF extract.

### 2.3. ZS Extract Bioactivity

#### 2.3.1. Antimutagenicity

Gene mutation has been recognized as an important step in cancer development. Therefore, in vitro antimutagenicity evaluation is generally performed as the first step in identifying potential anticancer agents [59]. The experimental results of the antimutagenicity evaluation of the ZS extracts are presented in Table 2; Table 3. TA98 and TA100 strains were treated with ZSF and ZSP extracts at different concentrations (312.5–2500 µg·plate^−1^), in the presence or absence of a metabolic enzyme system (S9 mix). Upon treatment with the ZS extracts in the presence of a mutagen, the colony reduction and inhibition rate increased in a concentration-dependent manner. In the absence of the S9 mix, both ZSF and ZSP extracts increased the mutation inhibition rate of TA98 and TA100 in a concentration-dependent manner, with the inhibitory capacity showing a similar trend. Specifically, the inhibitory capacity for TA100 was higher than that for TA98. In the presence of the S9 mix, both ZSF and ZSP extracts increased the mutation inhibition rate of TA98 and TA100 in a concentration-dependent manner, but their inhibitory capacities were significantly different. In TA98, 2500 µg·plate^−1^ ZSF showed similar inhibitory activity to 625 µg·plate^−1^ ZSP. Meanwhile, in TA100, 2500 µg·plate^−1^ ZSF showed similar inhibitory activity to 312.5 µg·plate^−1^ ZSP. In both TA98 and TA100, the mutation inhibition rate of ZSP was close to 100% at concentrations exceeding 625 µg·plate^−1^. Overall, ZSF and ZSP exhibit similar antimutagenicity against direct mutagens such as 4-nitroquinoline n-oxide (4nqo) or sodium azide (SA); however, ZSP exhibits higher antimutagenicity against indirect mutagens such as a benzo[a]pyrene (BaP) in the presence of the S9 mix than ZSF. Therefore, in the presence of the S9 mix, ZSP contains greater amounts of bioactive substances than ZSF or contains bioactive substances (e.g., anti-mutagens) that are absent in ZSF. Antimutagenic mechanisms mainly include the inactivation of the mutagen, inhibition of the metabolic activity of the mutagen, and inactivation of the activated mutagen [4]. To date, the antimutagenic activity of natural products has been widely documented. In particular, phenolic compounds of green tea have been reported to exhibit antimutagenic activity through different mechanisms, such as mutagenic metabolism interference and DNA protection against electrophilic mutagenic substances [4,60,61]. Epigallocatechin-3-gallate and gallic acid are nucleophiles that can eliminate electrophilic mutagens [62]. Phytochemicals such as catechin in green tea and resveratrol in grapes also demonstrate exceptional antimutagenic activity [63,64]. ZSP extract showed greater anti-mutagenicity activity than green tea in the Ames test [65]. These findings justify further exploration of the ZS-derived bioactive compounds that demonstrate antimutagenic activity. It has been reported that various metabolites may have antimutagenic ability against several types of mutagens [62]. In this study, ZSP exhibited superior antimutagenic activity compared to ZSF, which was attributed to its higher phenolic content (Table 4). Previous studies demonstrate that ZS-derived coumarins and phenolic substances may be contributed to its antimutagenic activity [21,25,26,27,28,66,67]. The present study is the first to measure and compare the antimutagenic activity of ZSF and ZSP extract. Our results provide fundamental data to unveil the antimutagenic mechanism of ZS extracts. Instrumental qualitative and quantitative analyses and further in vivo research are warranted.

#### 2.3.2. Antioxidant Activity

##### Total Polyphenol Content (TPC) and Total Flavonoid Content (TFC)

Phenolic compounds have garnered much interest as natural substances with bioactivity, particularly their antioxidant capacity. Phenolic hydroxyl group is a potent hydrogen donor and can react with ROS, terminating the new radical generation pathway [68]. Flavonoids are phenolics. The TPC and TFC of the ZS extracts from the calibration curve drawn based on the standard are shown in Table 4. The TPC of ZSP was higher than that of ZSF, whereas the TFC of ZSF was higher than that of ZSP. In a previous study, the TPC of ZS was in the range of 52.57–62.07 mg GAE·g^−1^, similar to the value obtained in the present study [69]. In addition, different phenolic compounds have been identified from ZS [34]. The present study is the first to analyze phenolics and flavonoids by comparing ZSF and ZSP, providing comprehensive data on the antioxidant activity of ZS extracts.

##### Free Radical Scavenging Activity

2,2-Diphenyl-1-picrylhydrazyl (DPPH) and 2,2′-azino-bis-3-ethylbenzothiazoline-6-sulfonic acid (ABTS) assays were performed to investigate the radical scavenging activity of ZSF and ZSP. The radical scavenging activity of the ZS extracts was expressed in terms of IC_50_ values calculated using linear regression analysis, and the results are shown in Table 5. In the DPPH assay, the IC_50_ value of ZSP was approximately four times smaller than that of ZSF but much greater than that of ascorbic acid (*p* < 0.05). In the ABTS assay, the IC_50_ value of ZSP was approximately two times smaller than that of ZSF but approximately two times greater than that of ascorbic acid (*p* < 0.05). The radical scavenging activity of the ZS ethanol extract measured in the present study was superior to that of the ZS oil extract reported in a previous study [70]. The antioxidant activity of ZS, including its hydroxyl and DPPH radical scavenging activity, has been reported [71]. The difference in radical scavenging activity between ZSP and ZSF may be related to the difference in their TPC (Table 4). Schinifoline, a 4-quinolinone derivative, was found in ZSP, and its radiosensitizing effect was investigated [24]. As quinolinone derivatives have strong antioxidant activity, the excellent antioxidant activity of ZSP may be due to the presence of schinifoline [72]. The test samples (ZSF, ZSP, and ascorbic acid) showed significant differences in the antioxidant activity, which was measured using the DPPH and ABTS assays. The DPPH and ABTS assays employ a similar principle to measure the non-physiological radical-scavenging ability of a sample. However, the ABTS assay measures the reduction of blue/green ABTS+ by antioxidants, while the DPPH assay measures the reduction of the purple DPPH to 1,1-diphenyl-2-picryl hydrazine [73]. The present study is the first to analyze the radical scavenging activity of ZSF and ZSP, providing comprehensive data on the antioxidant activity of ZS extracts.

#### 2.3.3. Anti-Inflammatory Activity

**Figure 6:**
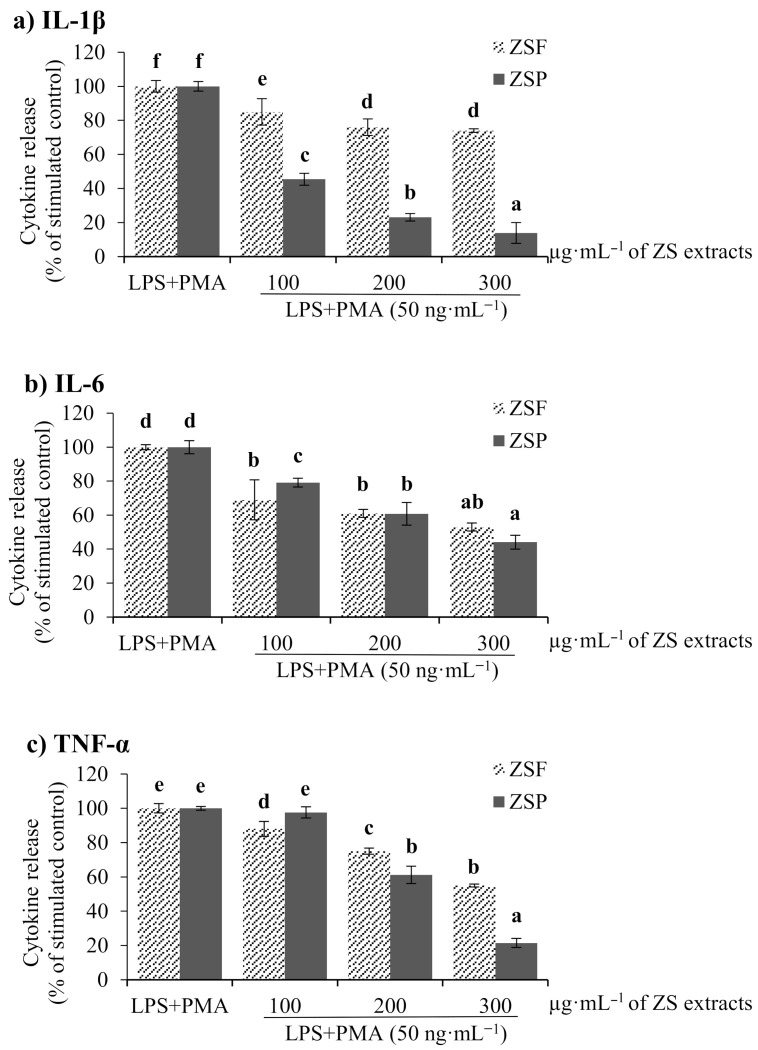
Anti-inflammatory effects of *Zanthoxylum schinifolium* extracts (ZSF: *Z. schinifolium* fruit extract, ZSP: *Z. schinifolium* pericarp extract) with the cytokines (**a**) IL-1β, (**b**) IL-6, and (**c**) TNF-α induced from Lipopolysaccharide (LPS) + phorbol 12-myristate 13-acetate (PMA). Data are expressed as the mean ± SD of triplicate experiments. ^a–f^ Within the graph, means with different letters are significantly different (Duncan’s multiple range test, *p* < 0.05).

When inflammation occurs due to external stimuli, cytokines such as IL-1β, IL-6, and TNF-α are released from inflammatory cells. These inflammatory mediators act on blood vessels or cells to promote inflammatory responses [17]. The inhibition of inflammatory mediators is effective in the treatment of inflammation. Accordingly, to evaluate the anti-inflammatory effects of the ZS extracts, the inhibition of cytokines, namely IL-1β, IL-6, and TNF-α, was assessed, and the results are presented in Figure 6. Lipopolysaccharide (LPS) and phorbol myristate acetate (PMA) are used as inflammagens, and the combination of LPS and PMA is commonly used to activate and induce the oxidative burst response in macrophages [74]. Inhibition of cytokine release was observed when the ZS extracts were treated with inflammagens. Both ZSF and ZSP inhibited IL-1β in a concentration-dependent manner, but there was no significant difference in the inhibition rates at 200 and 300 µg·mL^−1^ ZSF. The IC_50_ value of ZSP (134.4 ± 7.8) was approximately four times smaller than that of ZSF (537 ± 11.6). For IL-6, results similar to those for IL-1β were measured, but the IC_50_ value of ZSP (262.8 ± 11.2) was similar to that of ZSF (289.3 ± 5.5). TNF-α inhibited in a concentration-dependent manner, and the IC_50_ value of ZSP (223.8 ± 5.8) was approximately 1.5 times smaller than that of ZSF (349.3 ± 13.1). Our results indicate that ZSP is superior to ZSF in terms of inhibiting the three cytokines assessed. Therefore, ZSP likely contains greater amounts of bioactive substances than ZSF or contains bioactive anti-inflammatory substances that are absent in ZSF. In previous studies, the anti-inflammatory activities of ZS, including NO inhibition; iNOS protein inhibition; COX-2 protein inhibition; and TNF-α-induced VCAM-1, ICAM-1, and E-selectin protein inhibition, have been reported [32,75,76]. The present study is the first to measure the anti-inflammatory activities of ZSF and ZSP in terms of cytokine inhibition. Our results provide fundamental data to elucidate the anti-inflammatory mechanisms of ZS extracts. Nonetheless, instrumental analysis and further in vivo research are essential.

## 3. Materials and Methods

### 3.1. Plant Materials

*Zanthoxylum schinifolium* fruit (ZSF) and pericarp (ZSP) used in this experiment were purchased in the herbal medicine market located in Dongdaemun-gu, Seoul, Korea, and the dried materials were used after being ground into powder.

### 3.2. Chemicals/Reagents

Laboratory reagents including ethanol, water, Folin-Ciocalteu’s phenol reagent. gallic acid, quercetin, phosphate-buffered saline (PBS), neutral red, dimethyl sulfoxide (DMSO), benzo[a]pyrene (BaP), 4-nitroquinoline n-oxide (4nqo), sodium azide (SA), 2,2-diphenyl-1-picrylhydrazyl (DPPH), 2,2′-azino-bis-3-ethylbenzothiazoline-6-sulfonate (ABTS), agar, glucose, histidine/biotin, nutrient broth no. 2, NaCl, Lipopolysaccharide (LPS), and phorbol 12-myristate 13-acetate (PMA) were purchased from Sigma-Aldrich Co. (St. Louis, MO, USA). The co-factors for the NADH regenerating system and the S9 fraction of rat liver was purchased from Wako (Osaka, Japan) and Mol-Tox (Annapolis, MD, USA). EZ-CYTOX kit for cytotoxicity test is available from DoGenBio Co. (Seoul, Korea), and the ELISA kit for inflammation test was purchased from Abcam (Cambridge, UK). All other chemicals and reagents used were of analytical grade.

### 3.3. Strain and Cell/Medium

*Salmonella typhimurium* TA98 and TA100, the strains used in the mutagenicity test, were purchased from the Korea Research Institute of Bioscience and Biotechnology Gene Bank and were used mainly for testing while regularly checking their genetic traits. The WI-38 cell line (human lung normal cells) used for the cytotoxicity test and the U-937 cell line with macrophage characteristics used for the inflammation test were purchased from Korea Cell Line Bank (KCLB). The RPMI medium used for cell culture was Gibco, Thermo Fisher Scientific Inc. (Waltham, MA, USA), and Penicillin-Streptomycin was obtained from Thermo Fisher Scientific Inc. (Waltham, MA, USA), FBS was obtained from BioMed Co. (Gyeonggi-do, Korea).

### 3.4. Preparation of ZSF and ZSP Ethanol Extracts

Powder of ZSF (300 g) and ZSP (300 g) was soaked with 3 L of 70% ethanol (EtOH) at 22 °C for 24 h and then filtered. This process was repeated 3 times, and the filtrate was concentrated under reduced pressure using a rotary vacuum evaporator to obtain *Zanthoxylum schinifolium* (ZS) extracts and lyophilized. The obtained material was dissolved in DMSO and stored at −20 °C before being used in the experiment.

### 3.5. Safety Evaluations

#### 3.5.1. Strain Culture

The strains used for mutagenicity experiments were histidine-dependent (His-) mutation tester strains TA98 and TA100, and their genetic properties were tested regularly; uvrB mutation test, rfa (Δ) mutation test, histidine requirement test, R-factor identification test, and natural regression. For a 2.5% Nutrient broth solution as the culture medium of the strain, 3 g nutrient broth no.2 (Sigma-Aldrich Co., St. Louis, MO, USA) was added to 120 mL distilled water and autoclaved. Then, 30 µL and 20 µL of TA98 and TA100 strains are inoculated into 20 mL medium, respectively. The flask inoculated with the strain was operated in a rotator at 80 rpm, 37 °C, for 11 h. Cultured strains are refrigerated until used for experiment.

#### 3.5.2. Mutagenicity Assay

The mutagenicity of ZSF and ZSP was determined by the method described by Maron and Ames (1983) and OECD Guideline Test No. 471 (OECD 1997) in S. typhimurium strains TA98 and TA100 [77]. Test samples were integrated directly into the plate with or without metabolic enzyme system (S9 mix). As positive controls, 4NQO (1.0 µg·plate^−1^) and SA (1.0 µg·plate^−1^) were used as direct mutagens in the absence of S9 mix, and bap (2.0 µg·plate^−1^) was used as an indirect mutagen in the presence of S9 mix. DMSO was used as a vehicle control. Test samples diluted in DMSO were treated at different concentrations. (312.5~2500 µg·plate^−1^). A total of 100 µL of the cultured strain, 100 µL of the test sample, and 500 µL of S9 mix or PBS (Ph 7.4) were combined in 2 mL of top agar. Then, the composite tube was vortexed for 2–3 s and applied to a minimal glucose agar plate. When the applied composites solidified, the plate was closed and inverted. After the plates were incubated at 37 °C for 48 h, the number of revertant colonies to each plate was counted.

#### 3.5.3. Cytotoxicity Assay

##### Cell Culture

Cell lines (WI-38 and U-937; Korea Cell Line Bank, Seoul, Korea) were grown in RPMI media with 10% fetal bovine serum (FBS), 10,000 units·mL^−1^ of penicillin, and 10,000 units·mL^−1^ of streptomycin in an incubator (5% CO_2_) at 37 °C (Thermo Fisher Scientific Inc., Waltham, MA, USA). Cells were maintained for 2–3 days prior to passage and used for experiments at passages 5–20.

##### Neutral Red Uptake (NRU) Assay

To measure the cytotoxicity of ZS extracts in adherent WI-38 cells, the NRU assay was performed according to the procedure developed by Repetto et al. (2008) [78]. WI-38 cells were seeded with culture medium at a density of 2.0 × 10^4^ cells in per well in a 96-well plate, and incubated for 24 h in an incubator (5% CO_2_) at 37 °C. After aspiration of the culture medium, 200 µL of ZS extracts of different concentrations were added to each well, and incubated for 24 ± 2 h. Treatments and controls in each well were replaced with 200 µL neutral red solution and further incubated for 3 h. The neutral red solution was aspirated and 200 µL wash-fix solution (1% formalin solution; Biosesang, Gyeonggi-do, Korea) was added to each well for 1 min. Finally, after the wash-fix solution was aspirated, 200 µL neutral red extraction solution (50% ethanol containing 1% acetic acid) was added to each well. The plate was shaken for 1 min on a microplate shaker. The optical density (O.D) value of each well was measured at a wavelength of 540 nm using a microplate reader (Thermo Scientific, Vantaa, Finland). The measurements were expressed as a percentage of the control group. IC_50_ values (the concentration of sample required to inhibit 50% of cell growth) were calculated by linear regression analysis.

##### WST Assay

The EZ-CYTOX kit (DoGenBio, Seoul, Korea) was used to measure the cytotoxicity of ZS extracts in U-937 cells, which are floating cells used for inflammation experiments. WST assay was performed according to the protocol provided by the place of purchase of the kit. U-937 cells were seeded with culture medium at a density of 2.0 × 10^4^ cells per well in a 96-well plate and incubated for 24 h. ZS extracts were added at 10 µL to each well, and cells were incubated for 24 ± 2 h. An amount of 10 µL EZ-Cytox was added to each well and incubated for 3 h. The plate was shaken for 1 min on a microplate shaker, and the optical density (OD) value of each well was measured at a wavelength of 450 nm using a microplate reader. The measurements were expressed as a percentage of the control group. IC_50_ values (the concentration of sample required to inhibit 50% of cell growth) were calculated by linear regression analysis.

#### 3.5.4. Measurement of Intracellular ROS Generation

ROS production of ZS extracts was measured in WI-38 cells using the DCFDA Cellular ROS Detection Assay Kit (Abcam, Cambridge, UK). WI-38 cells (2.5 × 10^4^ cells per well) were seeded in 96-well black plates and incubated for 24 h. After aspiration of the culture medium, cells were treated with 100 µL 25 µM DCF-DA solution and incubated for 45 min. DCF-DA solution was aspirated, and 100 µL of test substances were added to each well and incubated for 6 h. The fluorescence spectrum was measured using a fluorescence reader (Biotek, Winooski, VT, USA) at the excitation and emission wavelengths of 485 and 535 nm, respectively. The measurements were expressed as a relative value compared to the vehicle control.

### 3.6. Evaluation of Bioactivity of ZS Extracts

#### 3.6.1. Antimutagenicity of ZS Extracts

The antimutagenicity assay was performed on the same basis as the mutagenicity assay described above. A total of 100 µL of cultured strain, 50 µL of test sample, 50 µL of mutagen and 500 µL of S9 mix or PBS (Ph 7.4) were combined in 2 mL of top agar. Plates coated with the complexes were incubated at 37 °C for 48 h, and then the number of colonies in each plate was counted. The mutation inhibition rate was calculated as follows.

Inhibition (%) = (M − B)/(M − A) × 100(1)

where M = number of revertant colonies in the presence of only mutagen, A = number of spontaneous revertant colonies, B = number of revertant colonies in the presence of both mutagen and the sample.

#### 3.6.2. Antioxidant Activity of ZS Extracts

##### Total Polyphenol Content

The total phenol content of ZSF and ZSP was determined according to modification of the method described by Dewanto et al. (2002) [79]. A total of 50 µL 2 N Folin-Ciocalteu’s phenol reagent was added to each 100 µL sample (1 mg·mL^−1^) and incubated for 10 min at room temperature. A total of 300 µL 20% Na2CO3 was added to the solutions and incubated for 15 min. The mixtures were mixed with 1 mL of water, and the final reactants were dispensed at 200 µL/well in a 96-well plate and absorbance was measured at 745 nm by a microplate reader. A calibration curve was drawn using gallic acid as a standard, and ZS extracts were expressed mg of equivalent gallic acid (GAE) per 1 g of sample.

##### Total Flavonoid Content

The total flavonoid content of ZSF and ZSP was determined according to modification of the method described by Pourmorad et al. (2006) [80]. A total of 100 µL 10% aluminum nitrate and 100 µL 1 M potassium acetate were added to 500 µL of each extract (1 mg·mL^−1^), and 4.3 mL 80% methanol was mixed. The mixtures were incubated at room temperature for 40 min, and then absorbance was measured at 420 nm by a microplate reader. A calibration curve was drawn using quercetin as a standard, and ZS extracts were expressed mg of equivalent quercetin (QE) per 1 g of sample.

##### DPPH Radical Scavenging Ability

The DPPH free radical scavenging ability was measured by modifying the method described by Blois (1958) [81]. Then, 200 µL samples were added to 800 µL 0.2 µM DPPH solution (in methanol), After 30 min dark reaction, absorbance was measured at 517 nm by a microplate reader. L-ascorbic acid was used as a reference material and positive control. The percentage of DPPH radical scavenging activity was calculated using the formula:

DPPH radical scavenging activity (%) = [1 − (OD of sample/OD of blank)] × 100(2)

IC_50_ values (the concentration of sample required to inhibit 50% of DPPH radicals) were calculated by linear regression analysis.

##### ABTS Radical Scavenging Ability

The ABTS free radical scavenging ability was measured by modifying the method described Re et al. (1999) [82]. A total of 50 mL 7 mM ABTS solution with 0.88 mL 2.6 mM potassium persulfate was incubated overnight in the dark to form ABTS cations. The solution was diluted with 5 mM sodium phosphate buffer until an absorbance of 0.7 ± 0.02 was obtained at a wavelength of 734 nm. Then, 0.1 mL samples were added to an appropriately diluted 1 mL ABTS solution and reacted for 10 min in the dark. Absorbance was measured at 734 nm by a microplate reader. The percentage of ABTS radical scavenging activity was calculated using the formula:

ABTS radical scavenging activity (%) = [1 − (OD of sample/OD of blank)] × 100(3)

IC_50_ values (the concentration of sample required to inhibit 50% of ABTS radicals) were calculated by linear regression analysis.

#### 3.6.3. Anti-Inflammatory Activity of ZS Extract through ELISA Kit

ELIISA kit (Abcam, Cambridge, UK) was used to examine the anti-inflammatory activity of ZS extracts, and the experiment was performed according to the protocol provided by the place of purchase of the kit. U-937 cells were seeded with a culture medium at a density of 2.0 × 10^4^ cells per well in a 6-well plate, and 2 mL of samples were added to each well. This plate was incubated for 3 h, then centrifuged (13 g, 15 min, 4 °C), and used for experiments. IL-1 beta, IL-6, and TNF-α were used as standards and were appropriately diluted. Standards or samples were incubated for 3 h simultaneously with biotinylated monoclonal antibodies specific for each antibody. The enzyme streptavidin-HRP that binds to the biotinylated antibody was added and incubated for 30 min. TMB substrate solution was added and incubated in the dark at room temperature for 10–20 min. After the stop reagent was added to each well, the absorbance was measured at 450 nm by a microplate reader immediately. The measurements were expressed as a percentage of the control group. IC_50_ values (the concentration of sample required to inhibit 50% of cytokine) were calculated by linear regression analysis.

### 3.7. Statistical Analysis

All results of experiments were expressed as mean ± standard deviation (SD). All analyses were calculated using the IBM Statistical Package for the Social Sciences 22 (SPSS Inc., Chicago, IL, USA). Results for all values obtained using one-way analysis of variance (ANOVA) were compared. Dunkan’s multiple comparison tests were used for pairwise comparisons. Values of *p* < 0.05 were considered statistically significant.

## 4. Conclusions

Our study provides a baseline assessment of the safety and bioactivity of ZSF and ZSP extracts. In the present study, the safety of the ZS extracts, including their mutagenicity, cytotoxicity, and intracellular ROS production, was investigated. Specifically, the mutagenicity of ZSP and ZSF was tested for the first time. Furthermore, the bioactivity of the ZS extracts was evaluated, and nontoxic concentration ranges were obtained through safety evaluation. The present study is the first to compare the bioactivities of ZSF and ZSP. ZSP is superior to ZSF in terms of antimutagenic, antioxidant, and anti-inflammatory activities. This may be attributed to the observed differences in the phenolic content between ZSF and ZSP extracts, as well as differences in other phytochemicals that are abundant in ZS, such as coumarins and alkaloids. We propose that research and development of pharmaceuticals and functional foods may benefit more from ZSP alone rather than the combined use of ZSF and ZSP. Furthermore, the identification of bioactive ingredients, as well as the evaluation of toxicity, metabolism, bioavailability, and other biological effects, should be the focus in future in vivo studies regarding ZSP.

## Figures and Tables

**Table 1:** Yield of each *Zanthoxylum schinifolium* extract.

Sample	Yield (%) ^1^
ZSF	10.96
ZSP	25.58

^1^ Yield (%) = extract powder (g)/dry raw material (g) × 100; ZSF: *Z. schinifolium* fruit extract, ZSP: *Z. schinifolium* pericarp extract.

**Table 2:** Antimutagenic effects of *Zanthoxylum schinifolium* extracts against mutagens in *Salmonella typhimurium* TA98, with or without the S9 mix.

Treatment	Concentration (µg·plate^−1^)	− S9		+ S9	
		Reversion ^2^	Inhibition (%) ^3^	Reversion	Inhibition (%)
Control ^1^	0	27 ± 3	-	33 ± 6	-
	1	476 ± 47	-	289 ± 7	-
ZSF	312.5	449 ± 6	6.0 ^c^	255 ± 23	13.3 ^e^
	625	433 ± 11	9.6 ^b,c^	228 ± 18	23.8 ^d^
	1250	412 ± 21	14.2 ^b^	107 ± 14	71.1 ^c^
	2500	338 ± 29	30.7 ^a^	37 ± 2	98.4 ^a^
ZSP	312.5	476 ± 24	0.0 ^d^	57 ± 11	90.6 ^b^
	625	449 ± 3	6.0 ^c^	39 ± 6	97.7 ^a^
	1250	392 ± 12	18.7 ^b^	32 ± 1	100.4 ^a^
	2500	322 ± 4	34.3 ^a^	32 ± 9	100.4 ^a^

Data are expressed as the mean ± SD of triplicate experiments. ZSF: *Z. schinifolium* fruit extract, ZSP: *Z. schinifolium* pericarp extract. ^1^ Control: 0: Number of spontaneous His+ Revertant colonies, 1: Number of direct mutagen-induced (1 µg·plate^−1^ 4NQO without the S9 mix, 1 µg·plate^−1^ BaP with the S9 mix) revertant colonies. ^2^ Number of revertant colonies. ^3^ Inhibition (%) = (Revertant colonies in control [mutagen]—Revertant colonies in sample)/(Revertant colonies in control [mutagen]—Spontaneous revertant colonies) × 100. ^a–e^ Within the column, means with different letters are significantly different (Duncan’s multiple range test, *p* < 0.05).

**Table 3:** Antimutagenic effects of *Zanthoxylum schinifolium* extracts against mutagens in *Salmonella typhimurium* TA 100, with or without the S9 mix.

Treatment	Concentration (µg·plate^−1^)	− S9		+ S9	
		Reversion ^2^	Inhibition (%) ^3^	Reversion	Inhibition (%)
Control ^1^	0	124 ± 7	-	146 ± 8	-
	1	412 ± 13	-	437 ± 42	-
ZSF	312.5	336 ± 6	26.4 ^b,c^	413 ± 25	8.2 ^d^
	625	322 ± 32	31.2 ^b^	401 ± 40	12.4 ^d^
	1250	263 ± 28	51.7 ^a^	370 ± 23	23.0 ^c^
	2500	253 ± 32	55.2 ^a^	191 ± 38	84.5 ^b^
ZSP	312.5	386 ± 20	9.0 ^d^	188 ± 25	85.6 ^b^
	625	375 ± 34	12.8 ^d^	157 ± 7	96.2 ^a^
	1250	354 ± 8	20.1 ^c^	148 ± 11	99.3 ^a^
	2500	254 ± 34	54.9 ^a^	147 ± 2	99.7 ^a^

Data are expressed as the mean ± SD of triplicate experiments. ZSF: *Z. schinifolium* fruit extract, ZSP: *Z. schinifolium* pericarp extract. ^1^ Control: 0: Number of spontaneous His+ Revertant colonies, 1: Number of indirect mutagen-induced (1 µg·plate^−1^ SA without the S9 mix, 1 µg·plate^−1^ BaP with the S9 mix) revertant colonies. ^2^ Number of revertant colonies. ^3^ Inhibition (%) = (Revertant colonies in control [mutagen] − Revertant colonies in sample)/(Revertant colonies in control (mutagen) − Spontaneous revertant colonies) × 100. ^a–d^ Within the column, means with different letters are significantly different (Duncan’s multiple range test, *p* < 0.05).

**Table 4:** Total phenolic content and total flavonoid content of *Zanthoxylum schinifolium* extracts.

Sample	Total Phenol Content (mg GAE·g^−1^) ^1^	Total Flavonoid Content (mg QE·g^−1^) ^2^
ZSF	22.7 ± 3.1 ^a^	18.6 ± 2.5 ^b^
ZSP	62.3 ± 7.9 ^b^	10.8 ± 2.0 ^a^

Data are expressed as means ± SD from triplicate experiments. ZSF: *Z. schinifolium* fruit extract, ZSP: *Z. schinifolium* pericarp extract. ^1^ Total phenol content is expressed as gallic acid equivalent (GAE) in milligram per gram of sample. ^2^ Total flavonoid content is expressed as quercetin equivalent (QE) in milligram per gram of sample. ^a,b^ Within the column, means with different letters are significantly different (Duncan’s multiple range test, *p* < 0.05).

**Table 5:** 2,2-Diphenyl-1-picrylhydrazyl (DPPH) and 2,2′-azino-bis-3-ethylbenzothiazoline-6-sulfonic acid (ABTS) radical scavenging activity of *Zanthoxylum schinifolium* extracts.

Sample	DPPH Radical Scavenging Activity	ABTS Radical Scavenging Activity
	IC_50_ (µg·mL^−1^) ^1^	IC_50_ (µg·mL^−1^)
l-Ascorbic acid	4.1 ± 1.7 ^a^	22.9 ± 4.3 ^a^
ZSF	281.1 ± 16.8 ^c^	101.7 ± 11.5 ^c^
ZSP	75.6 ± 6.1 ^b^	57.4 ± 6.4 ^b^

Data are expressed as the mean ± SD of triplicate experiments. ZSF: *Z. schinifolium* fruit extract, ZSP: *Z. schinifolium* pericarp extract. ^1^ IC50 value is the concentration of sample required to inhibit 50% of DPPH and ABTS radicals. ^a–c^ Within the column, means with different letters are significantly different (Duncan’s multiple range test, *p* < 0.05).

## Data Availability

The data presented in study are available on request from the corresponding author.

## Sample Availability

Samples of the compounds are not available from the authors.
